# A novel heterotaxy gene: Expansion of the phenotype of *TTC21B*‐spectrum disease

**DOI:** 10.1002/ajmg.a.62093

**Published:** 2021-02-05

**Authors:** Alanna Strong, Dong Li, Frank Mentch, Hakon Hakonarson

**Affiliations:** ^1^ Division of Human Genetics Children's Hospital of Philadelphia Philadelphia Pennsylvania USA; ^2^ The Center for Applied Genomics Children's Hospital of Philadelphia Philadelphia Pennsylvania USA; ^3^ Department of Pediatrics, Perelman School of Medicine University of Pennsylvania Philadelphia Pennsylvania USA; ^4^ Division of Pulmonary Medicine Children's Hospital of Philadelphia Philadelphia Pennsylvania USA

**Keywords:** cilia, ciliopathy, heterotaxy, IFT139, TTC21B

## Abstract

*TTC21B* encodes the protein IFT139, a critical component of the retrograde transport system within the primary cilium. Biallelic, pathogenic *TTC21B* variants are associated with classic ciliopathy syndromes, including nephronophthisis, Jeune asphyxiating thoracic dystrophy, and Joubert Syndrome, with ciliopathy‐spectrum traits such as biliary dysgenesis, primary ciliary dyskinesia, and situs inversus, and also with focal segmental glomerulosclerosis. We report a 9‐year‐old male with focal segmental glomerulosclerosis requiring kidney transplant, primary ciliary dyskinesia, and biliary dysgenesis, found by research‐based exome sequencing to have biallelic pathogenic *TTC21B* variants. A sibling with isolated heterotaxy was found to harbor the same variants. This case highlights the phenotypic spectrum and unpredictable manifestations of *TTC21B*‐related disease, and also reports the first association between *TTC21B* and heterotaxy, nominating *TTC21B* as an important new heterotaxy gene.

## 1 INTRODUCTION

The primary cilium is a solitary organelle that exists atop most cell types and coordinates organ growth and patterning through its role as a chemosensory organelle and signaling hub. Dysfunction of the primary cilium is associated with ciliopathy syndromes, a group of disorders characterized by severe developmental defects of the brain, eye, heart, lungs, liver, kidney, and skeleton. Over 100 genes have been implicated in ciliopathies, including genes involved in ciliogenesis, ciliary signaling, ciliary transport, and ciliary maintenance (Badano, Mitsuma, Beales, & Katsanis, 2006; Shaheen et al., 2016). One such gene, tetratricopeptide repeat domain 21B (*TTC21B*), maps to chromosome 2q24.3, and encodes the protein intraflagellar transport protein 139 (IFT139), a critical component of ciliary retrograde transport (Tran et al., 2008). Biallelic variants in *TTC21B* are associated with classical ciliopathy syndromes including nephronophthisis, Jeune asphyxiating thoracic dystrophy, and Joubert Syndrome, with ciliopathy spectrum traits such as biliary dysgenesis, primary ciliary dyskinesia, and situs inversus, and also with focal segmental glomerulosclerosis (FSGS) (Davis et al., 2011; Huynh Cong et al., 2014; Zhang, Su, et al., 2018; Zhang, Taylor, et al., 2018). *TTC21B* is also a modifier gene for other ciliopathy syndromes, with heterozygous *TTC21B* variants exacerbating disease severity in human and animal models of ciliary disease (Davis et al., 2011).

More than 50 disease‐causing variants have been reported in *TTC21B*, including a founder variant (c.626C>T; p.(Pro209Leu)), which has been reported in the homozygous state in isolated FSGS and also in FSGS with extrarenal manifestations (Huynh Cong et al., 2014; Zhang, Su, et al., 2018). The recurrent p.(Pro209Leu) allele is a well‐studied hypomorphic allele; however, many reported *TTC21B* variants are true null alleles and can cause severe syndromic ciliopathies (Davis et al., 2011; Zhang, Su, et al., 2018).

Another class of ciliopathy syndromes, heterotaxy‐spectrum disease, is characterized by defects in organ placement and orientation due to impaired left–right patterning during embryogenesis (Sempou & Khokha, 2019; Shinohara & Hamada, 2017). Common associations include intestinal malrotation, asplenia or polysplenia, midline liver and stomach, and complex congenital heart disease with misplacement of the great vessels and abnormal ventricle morphology (Belmont, Mohapatra, Towbin, & Ware, 2004; Wallmeier et al., 2020). Additional comorbidities can include primary ciliary dyskinesia, biliary dysgenesis, cystic kidney disease, and nephronophthisis (Liu et al., 2020; Shapiro, Tolleson‐Rinehart, Zariwala, Knowles, & Leigh, 2015; Wallmeier et al., 2020). Genes implicated in heterotaxy‐spectrum disease typically encode proteins of the motile or moving cilia of the embryonic node, which beat in a rotary fashion to facilitate the distribution of developmental morphogens and the establishment of the left–right axis (Desgrange, Le Garrec, & Meilhac, 2018; Li et al., 2015; Shinohara & Hamada, 2017). Point mutations in established heterotaxy genes as well as copy number variations have both been described in heterotaxy‐spectrum disease (Belmont et al., 2004; Cowan et al., 2016; Gabriel, Young, & Lo, 2020; Sempou & Khokha, 2019). Although many genes have been implicated in left–right patterning and heterotaxy‐spectrum disease, the genetic landscape of these disorders is incompletely understood.

We report a family identified through research testing with multiple family members affected by ciliopathy‐spectrum disease found by research‐based exome sequencing to harbor biallelic *TTC21B* variants. These cases highlight the phenotypic diversity of *TTC21B*‐spectrum disease and report a novel association between *TTC21B* gene variants and heterotaxy.

### 1.1 Patient presentation

**FIGURE 1:**
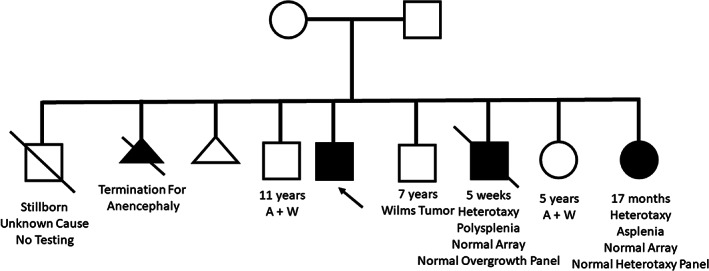
Pedigree for proband's family. Note stillborn male sibling, sibling with anencephaly, male sibling with total anomalous pulmonary venous return, unbalanced atrioventricular canal, midline liver, hepatomegaly, dilated renal pelvis, and polysplenia, and a female sibling with right atrial isomerism, hypoplastic left ventricle, pulmonary atresia, bilateral superior vena cavas, asplenia, heterotaxy‐spectrum, and developmental delay

Patient is a 9‐year‐old male with a history of FSGS status post kidney transplantation, biliary dysgenesis, and primary ciliary dyskinesia. He was born full term via vaginal delivery to a 26‐year‐old G5P2➔3 mother after an uncomplicated pregnancy. He did well until 3 years of age when he presented to his Pediatrician for persistent cough and was noted to have an elevated blood pressure, which persisted with follow‐up. A comprehensive metabolic panel was notable for an elevated creatinine and elevated aminotransferases. Renal ultrasound was notable for bilaterally small kidneys with increased cortical echogenicity. Echocardiogram showed a structurally normal heart with no evidence of left ventricular hypertrophy. Kidney biopsy showed tubular atrophy with interstitial fibrosis and inflammation as well as C1q deposits, consistent with a diagnosis of FSGS and C1q nephropathy. Liver biopsy showed mild portal fibrosis and biliary dysgenesis. Liver function and aminotransferases remained stable; however, renal function rapidly worsened, and he ultimately required kidney transplantation at 4 years of age. Other complications included myopia and hypertension managed with monotherapy. At 7‐years of age he was evaluated by Pulmonology for persistent cough. A chest X‐ray showed no skeletal abnormalities, but was notable for right middle and lower lobe atelectasis, and a chest CT was notable for right middle and lower lobe bronchiectasis with mucus plugging. He was formally diagnosed with primary ciliary dyskinesia and was started on a pulmonary clearance regimen with resolution of his cough. There have been no developmental concerns. Family history was notable for a stillborn male infant, an early termination for anencephaly, a brother with total anomalous pulmonary venous return, unbalanced atrioventricular canal, midline liver, hepatomegaly, dilated renal pelvis, and polysplenia with nondiagnostic microarray and overgrowth panel results who died at 5‐weeks of age, a 17‐month‐old sister with right atrial isomerism, hypoplastic left ventricle, pulmonary atresia, bilateral superior vena cavas, asplenia, heterotaxy‐spectrum and developmental delay with normal microarray and heterotaxy gene panel results, and a 7‐year‐old brother with a history of Wilms tumor (Figure 1). Of note, though both parents are of Ashkenazi‐Jewish descent, there is no known history of consanguinity, and microarray performed on the deceased brother with heterotaxy did not report any regions of homozygosity.

Patient was enrolled in a gene discovery research study through the Center for Applied Genomics (CAG) at The Children's Hospital of Philadelphia (IRB protocol # 16‐013278). Exome sequencing was performed, and revealed biallelic variants in *TTC21B*: a likely pathogenic splice variant (c.1088‐1G>C) and the founder pathogenic missense variant (c.626C>T; p.(Pro209Leu)). Patient's 17‐month‐old sister was subsequently evaluated clinically and was confirmed to carry the same pathogenic variants. Comprehensive metabolic panel and abdominal ultrasound were performed and were normal. There are no respiratory symptoms at this time. DNA could not be obtained for genetic testing for the deceased brother with heterotaxy‐spectrum disease nor from the sibling with anencephaly. All living siblings were tested for both familial variants and found to carry only the splice variant (unaffected brother and sister) or neither familial variant (brother with Wilms tumor).

## 2 DISCUSSION

*TTC21B* encodes the protein IFT139, a critical component of the retrograde ciliary transport system required for proper formation and function of the primary cilium. Its deficiency is associated with ciliopathy‐spectrum disease as well as FSGS (Davis et al., 2011; Huynh Cong et al., 2014; Zhang, Taylor, et al., 2018; Zhang, Su, et al., 2018). The extrarenal phenotype of *TTC21B*‐related disease is broad, most commonly including hypertension and myopia; however, other systemic features have been reported, including developmental delay, cerebral aneurysm, neural tube defect, hearing loss, situs inversus, recurrent pulmonary infections, restrictive lung disease, hepatic fibrosis, elevated aminotransferases, thoracic dystrophy, brachydactyly, and neutropenia (Davis et al., 2011; Zhang, Su, et al., 2018). Interestingly, though *TTC21B* is expressed in the retina and ciliopathy‐spectrum disease is often associated with retinal disease, there is inconsistent evidence of a retinal phenotype with *TTC21B* gene variants (Davis et al., 2011).

More than 50 disease‐causing *TTC21B* variants have been reported, including a common founder allele p.(Pro209Leu) in European and North African populations (Davis et al., 2011; Huynh Cong et al., 2014). Functional studies in human and cellular models suggest that the p.(Pro209Leu) variant is a hypomorphic allele, likely explaining why its homozygosity is typically associated with isolated FSGS or nephronophthisis, and not with syndromic ciliopathy (Huynh Cong et al., 2014). Additional genotype–phenotype correlations have emerged, suggesting that the p.(Pro209Leu) allele *in trans* with a nonsense or frameshift null allele is sufficient to cause a syndromic ciliopathy, and that the combination of nonsense and splice variants invariably produces severe disease such as Jeune asphyxiating thoracic dystrophy or early nephronophthisis with prominent extrarenal manifestations (Davis et al., 2011). Cellular and animal models also support a correlation between more deleterious *TTC21B* variants and increasing disease severity: siRNA‐mediated *TTC21B* knockdown in mIMCD3 cells results in severe structural ciliary differences, and morpholino‐mediated *ttc21b* knockdown in zebrafish causes gastrulation defects (Davis et al., 2011). The combination of the common hypomorphic p.(Pro209Leu) variant in our patient and his sister *in trans* with a splice‐altering variant likely explains their disease severity and prominent extrarenal manifestations.

Though situs inversus has been reported in *TTC21B‐*related disease, heterotaxy‐spectrum disease is a new association, and *TTC21B* is not included in commercial heterotaxy gene panels. Interestingly, heterozygous copy number variations involving *TTC21B* have been identified in a cohort of heterotaxy and congenital heart disease patients, supporting a role for this gene in the pathogenesis of heterotaxy (Cowan, 2015). It is tempting to speculate that *TTC21B* is a novel component of the nodal cilium that facilitates left–right patterning during embryogenesis.

The etiology for the phenotypic diversity in this family is unclear. Although not able to be genetically confirmed, it is likely that the fetus affected by anencephaly carried the familial biallelic *TTC21B* variants, as *TTC21B* is associated with neural tube defects. Additionally, the infant who died at 5‐weeks of age with complex congenital heart disease, midline liver, and polysplenia also likely carried both familial variants. Our proband has a structurally normal heart, but has kidney, liver, and lung disease, whereas his sister has no evidence of liver, kidney, or lung disease but has severe cardiac malformations. It is possible that she will go on to develop these extracardiac manifestations, and that her heart disease is related to additional variants in other ciliary genes exacerbating her phenotype, which is a well‐described feature of ciliopathy syndromes (Shaheen et al., 2016). In reference to our proband, careful interrogation of 101 known ciliopathy genes (Table S1) revealed no variants capable of explaining the severity of the proband's extracardiac manifestations compared with his siblings.

Here we present a novel association between biallelic *TTC21B* variants and heterotaxy‐spectrum disease as well as an example of extremely divergent ciliopathy phenotypes within a single family ranging from anencephaly to heterotaxy to multiorgan syndromic ciliopathy, and suggest that *TTC21B* be considered on the differential for children presenting with heterotaxy‐spectrum disease and situs abnormalities.

## CONFLICT OF INTEREST

The authors declare no conflicts of interest.

## Supporting information


**Table S1** Ciliopathy genes specifically interrogated in proband on exome sequencing

## Data Availability

Data sharing is not applicable to this article as no new data were created or analyzed in this study.
