# Supplementary material for: A novel heterotaxy gene: Expansion of the phenotype of TTC21B‐spectrum disease
Source: Am J Med Genet A. 2021 Feb 5;185(4):1266–9. doi: 10.1002/ajmg.a.62093 (PMC9290470; doi:10.1002/ajmg.a.62093)
Supplement: Supplementary file 1 — Table S1 Ciliopathy genes specifically interrogated in proband on exome sequencing [file AJMG-185-1266-s001.docx]

**Supplementary Table One**: Ciliopathy genes specifically interrogated in proband on exome sequencing

| AHI1 | BBS9 | CEP164 | DNAH8 | IFT172 | NEK8 | RSPH4A | TTC8 |
| --- | --- | --- | --- | --- | --- | --- | --- |
| ANKS6 | C21org59 | CEP290 | DNAI1 | IFT80 | NME8 | RSPH9 | WDPCP |
| ARL13B | C5orf42 | CEP41 | DNAI2 | INPP5E | NPHP1 | SDCCAG8 | WDR19 |
| ARL6 | CC2D2A | CEP83 | DNAL1 | INVS | NPHP3 | SPAG1 | WDR34 |
| ARMC4 | CCDC103 | CSPP1 | DRC1 | IQCB1 | NPHP4 | TCTN1 | WDR35 |
| B9D1 | CCDC114 | DCDC2 | DYNC2H1 | KIAA0586 | OFD1 | TCTN2 | WDR60 |
| B9D2 | CCDC151 | DNAAF1 | DYX1C1 | KIF7 | PDE6D | TCTN3 | XPNPEP3 |
| BBS10 | CCDC39 | DNAAF2 | EVC | LRRC6 | PKD2 | TMEM138 | ZMYND10 |
| BBS12 | CCDC40 | DNAAF3 | EVC2 | MCIDAS | PKHD1 | TMEM216 | ZNF423 |
| BBS2 | CCDC65 | DNAAF5 | GAS8 | MKKS | RPGR | TMEM231 |  |
| BBS4 | CCNO | DNAH1 | GLIS2 | MKS1 | RPGRIP1L | TMEM237 |  |
| BBS5 | CEP104 | DNAH11 | IFT122 | MRE11 | RSPH1 | TMEM67 |  |
| BBS7 | CEP120 | DNAH5 | IFT140 | NEK1 | RSPH3 | TRIM32 |  |
